# Psychometric validation techniques applied to the IND-VFQ-33 visual function questionnaire: the Hyderabad ocular morbidity in the elderly study (HOMES)

**DOI:** 10.1186/s12874-021-01217-w

**Published:** 2021-02-05

**Authors:** William Mitchell, Srinivas Marmamula, Nazlee Zebardast, Weiwen Ng, Joseph J. Locascio, Thirupathi Kumbam, Satya Brahmanandam, Navya Rekha Barrenkala

**Affiliations:** 1 Massachusetts Eye and Ear Infirmary, Department of Ophthalmology, Boston, USA; 2 Harvard TH Chan School of Public Health, Harvard University, Boston, USA; 3 Allen Foster Community Eye Health Research Centre, LV Prasad Eye Institute, Hyderabad, India; 4 Brien Holden Institute of Optometry and Vision Science, LV Prasad Eye Institute, Hyderabad, India; 5 Department of Biotechnology/Wellcome Trust India Alliance, LV Prasad Eye Institute, Hyderabad, India; 6 School of Optometry and Vision Science, University of New South Wales, Kensington, Australia; 7 Department of Ophthalmology, Harvard Medical School, Boston, USA; 8 University of Minnesota School of Public Health, University of Minnesota, Minneapolis, USA; 9 Department of Neurology, MA General Hospital, Boston, USA

**Keywords:** Ophthalmology, Psychometric validation, Factor analysis, Item response theory, Differential item functioning

## Abstract

**Background:**

Over 2 billion people suffer from vision impairment or blindness globally, and access to validated visual measurement tools in imperative in accurately describing and managing the burden of eye disease. The present study applies contemporary psychometric validation techniques to the widely used 33-item Indian Visual Function Questionnaire (IND-VFQ-33).

**Methods:**

We first estimated the polychoric correlation between each pair of items. Next, an unrotated and oblique Promax rotated factor analysis, item response theory (IRT, using a graded response model (GRM)), and differential item functioning (DIF) testing were applied to the IND-VFQ-33. We subsequently propose a validated IND-VFQ-33 questionnaire after psychometric testing, data reduction, and adjustment.

**Results:**

Exploratory unrotated factor analysis identified two factors; one with a particularly high eigenvalue (18.1) and a second with a lower eigenvalue still above our threshold (1.1). A subsequent oblique Promax factor rotation was undertaken for a 2-factor solution, revealing two moderately correlated factors (+ 0.68) with clinically discrete item loadings onto either Factor 1 (21 items; collectively labelled “daily activities”) or Factor 2 (5 items; collectively labelled “bright lights”). IRT confirmed high item discrimination for all remaining items with good separation between difficulty thresholds. We found significant DIF on depression for six items in Factor 1 (all uniform DIF, except item 21 (non-uniform DIF) with no substantive difference in beta thresholds for any item and no substantive difference in expected individual or sum score, by depression at baseline. For Factor 2, only one item demonstrated significant uniform DIF on gender, similarly without major differences in beta thresholds or expected total score between gender at baseline. Consequently, no further item recalibration or reduction was undertaken after IRT and DIF analysis.

**Conclusion:**

Applying IRT and DIF validation techniques to the IND-VFQ-33 identified 2 discrete factors with 26 uniquely-loading items, clinically representative of difficulty performing daily activities and experiencing difficulty due to bright lights/glare respectively. The proposed modified scale may be useful in evaluating symptomatic disease progression or response to treatment in an Indian population.

## Background

Globally over 2 billion people suffer from vision impairment or blindness, half of which is either preventable or treatable [1, 2]. Cataract and uncorrected refractive error are the two most prominent causes of vision impairment worldwide, disproportionately impacting low-middle income countries and older age groups [2, 3] where the future burden of global blindness is predicted to most severely affect [4]. With globally aging populations [5], access to visual-measurement tools validated for use in older populations is imperative in accurately describing and managing the burden of eye disease [6].

The Indian Visual Function Questionnaire (IND-VFQ-33) is a 33-question survey originally psychometrically validated in 2004 by Gupta and colleagues on a population of 780 patients in India [6]. The questionnaire was reduced from an original sample of 45 questions (henceforth referred to as “items”), removing items if they demonstrated either > 5% missing values, high inter-item correlation > 0.80, or for failing convergence/discrimination testing. Gupta and colleagues concluded that 33 of the original 45 items reliably assessed three clinical domains (or dimensions): (i) visual symptoms, (ii) psychosocial impact, and (iii) general function [6]. In 2012, Gothwal and colleagues fit Rasch models to each of these 3 domains. Based on lack of fit to the model, they recommended deleting the visual symptoms and psychosocial impact scales. They argued that the general function scale exhibited multidimensionality, and that it should be divided into two scales measuring visual function and vision-related mobility impairments [7].

Psychometric validation is a statistical method used to identify the presence and nature of underlying “latent traits” being assessed by a questionnaire. The present study applies Factor Analysis, and more contemporary Item Response Theory (IRT) and Differential Item Functioning (DIF) psychometric validation techniques to evaluate the IND-VFQ-33, using data from 867 questionnaire-respondents either with visual impairment (primarily due to cataract or uncorrected refractive error), or with normal vision.

IRT represents a collection of modern psychometric validation techniques recommended for reporting patient outcomes, suitable for the analysis of questionnaires that measure a latent construct (i.e., vision-related psychosocial symptoms, function and quality of life), and for estimating individual participant scores on the latent construct, based on responses to the items thereafter [8]. The Graded Response Model (GRM) is a type of IRT model particularly well-suited for validation of such questionnaires [9], and is applied in the present study [7]. The GRM model is particularly suited to validating ordinal scale items (frequently used in health assessments) and being less constrained than other IRT models, provides an accurate reflection of the data [10, 11]. The IRT validation process should also involve an assessment of DIF [12]. DIF assesses if the item responses are different between levels of some baseline variable (usually sociodemographic, e.g. gender, ethnicity, age) though the estimated person latent score is constant, thus indicating the item is responsive to the extraneous baseline variable in addition to or instead of the person score, suggesting impure validity for assessing the latent variable of interest. If enough items exhibit DIF for any one sociodemographic group vs its counterpart, then the same raw score for that group might indicate a different level of visual impairment. This could compromise the ability to make screening or clinical decisions [13, 14]. In that case, clinicians might decide that some groups of respondents will need their questionnaire scores re-calibrated for accurate comparisons [8].

Prior psychometric validation techniques applied to visual questionnaires (for example, Rasch models) impose comparatively more restrictions than methods applied herein (for example, assuming equal discrimination of impairment levels for all items, rather than calculating discrimination levels). Such constraints have consequently caused other studies to remove entire sub-scales, and to substantively change the content of the general function scale based on violations of assumed unidimensionality [10, 11, 15]. We believe that the Rasch model’s restrictions may be unrealistic for such surveys, and that by fitting the more flexible GRM, we provide a more accurate reflection of the data.

The present study applies contemporary psychometric validation methods described above, which have not yet been applied to the IRT-VFQ-33. Questionnaire responses from a large residential aged-care population in India suffering eye diseases representative of the commonest causes of visual impairment in low income countries were used. We subsequently propose an adjusted, validated IND-VFQ-33 questionnaire after psychometric testing, data reduction and adjustment, appropriate for use particularly in an elderly residential care population.

## Methods

### Study design, population, and the IND-VFQ-33 questionnaire

The IND-VFQ-33 is a 33-question Rasch validated instrument developed and validated in India [6, 16], and assesses the visual dimensions of visual functioning and activity limitation, psychosocial impact, and visual symptoms in the three distinct sub-scales mentioned [7, 16, 17]. Questions 1–22 of the IND-VFQ-33 are scaled on a 5-point ordinal difficulty scale, and the remaining 11 questions scaled on a 4-point ordinal scale. Options 1–4 on both scales are identical in options reporting degree of difficulty; (1) “not at all”, (2) “a little”, (3) “quite a bit”, and (4) “a lot”; where items 1–22 also include a fifth difficulty option ((5) “cannot do this because of my sight”). Questions 1–22 also had a sixth option ((6) “cannot do this for other reasons”) which was treated as effectively a missing response. A higher score on the scale represents a higher degree of difficulty.

Participants from the HOMES study, originally conducted to assess the burden of vision loss in older adult populations in residential care in India, were considered for the present study cohort [18]. Participants were excluded if they demonstrated cognitive deficit (defined here as a Mini-Mental State Examination (MMSE) score of < 20), or for medical conditions precluding participation. Participants were categorised as having either normal vision (presenting visual acuity 6/18 or better in the better eye), or having significant visual impairment (classified as presenting visual acuity worse than 6/18 in the better eye) [2]. IND-VFQ-33 questionnaires were administered to participants by trained investigators [18]. The HOMES study design and procedures were approved by the Institutional Review Board of the Hyderabad Eye Research Foundation, India. The study was conducted in adherence to the Declaration of Helsinki. All participants provided written informed consent expressing their willingness to participate in the study.

### Unrotated and rotated (Promax oblique) factor analysis

Factor Analysis is a technique used to identify the presence and nature of latent traits underlying participant responses (where latent traits are unobservable characteristics (for example, experiencing visual difficulty in dim light)). Because IRT models assume only one latent trait influences responses to each question (potentially producing biased estimates of trait levels and item parameters if violated), an exploratory Factor Analysis is necessary to first analyze correlations between questions (henceforth referred to as “items”). This allows the number and nature of latent traits (henceforth referred to as “factors”) causing observed item responses to be determined [19–22], and the underlying basis for all their observed inter-correlations. Specifically, exploratory factor analysis analyzes the correlations of responses to items to identify unique factors, on the assumption that unique patterns of responses suggest which factors are likely being assessed, and which items relate to those factors (and to what degree) [23].

We initially estimated the pairwise polychoric correlation between each pair of items, which are essentially estimates of the correlations of hypothetical continuous variables corresponding respectively to each observed categorical or ordinal variable (presumably derived by binning the latent variable at cutoffs) and responsible for their surface relations. Factor Analysis is known to sometimes give distorted results when applied directly to correlations of numerically coded ordinal or categorical variables. Next, we conducted a Factor Analysis on the correlation matrix; this determines if the questionnaire is unidimensional (where a single factor is being measured by a collection of items), or multidimensional (where more than one factor underlies the various items). The criteria used to identify the number of factors are (i) eigenvalues (essentially factor variances) that are > = 1 (the variance of a standardized variable), (ii) a “scree” plot of eigenvalues, and/or (iii) a parallel analysis [21, 23]. The point at which factor variances show an “elbow” bend and asymptote to a floor in the “scree” plot suggests the number of factors. A parallel analysis compares each obtained factor’s eigenvalue to the 95th percentile of the distribution of their respective counterparts produced by random permutations of the data as a method of determining the statistical significance of each factor. The short-listed number of factors at this stage is then pre-specified in a subsequent factor analysis that is “rotated” to a statistically more parsimonious and hopefully more substantively meaningful solution in which the constellation of item loadings (associations) on each factor indicate the nature of the underlying latent construct and suggest a suitable corresponding label to describe it. We employed a type of “oblique” rotation method (Promax) that allowed factors to be moderately correlated if empirically indicated as such [24].

All items with > 20% missing values were removed from the final list of items and their factors. Items were also removed if they either (i) loaded poorly (< 0.5) on every factor identified in rotated factor analysis, and/or (ii) cross-loaded (i.e. loaded well on more than one factor identified) [25, 26].

### Item response theory (graded response models)

Item Response Theory (IRT) was undertaken next and involves fitting a latent variable model to item responses intended to measure (in this case) difficulty performing visual-related tasks [27]. Essentially, the aim of IRT modelling is to assess relative item difficulty, assess how well items discriminate between participants of differing ability (discrimination), and (by re-scaling responses in order of difficulty) calculate an ability-score (usually called theta) for each participant.

IRT models validate how well individual items discriminate between participants of differing estimated “ability”, and how clearly those differences in ability are reflected by individual item responses [28], using a difference model [29] which defines the cumulative probability among response options as:

$$ {\boldsymbol{P}}_{\boldsymbol{k}}^{\ast}=\boldsymbol{P}\left({\boldsymbol{x}}_{\boldsymbol{ip}}\boldsymbol{\ge}\boldsymbol{k}\ \right|\ {\boldsymbol{\theta}}_{\boldsymbol{p}}\Big) $$

Where the probability of responding ***k ≥*** **1** denoted by ${\boldsymbol{P}}_{\mathbf{1}}^{\ast}$ is exactly 1.0, because any observed response to an item must be in category 1 or higher [28]. The probability ${\boldsymbol{P}}_{\mathbf{2}}^{\ast}$ of responding ***k ≥*** **2** is then estimated from response data. Taking the difference between ${\boldsymbol{P}}_{\mathbf{2}}^{\ast}$ and ${\boldsymbol{P}}_{\mathbf{1}}^{\ast}$ leaves category ***k =*** **1** in isolation. By creating a series of dichotomous probabilities in the same step-wise manner, we can model the response function of each category up to ***k =*** **5** [28].

The specific class of IRT difference model used for the present study, the GRM, was originally developed by Samejima in 1969 [9]. The GRM applies the above principles of traditional dichotomous unidimensional IRT models to ordinal data (like the IND-VFQ-33) [9, 28], by calculating a series of dichotomous probabilities for each option on the polytomous 4- or 5-point ordinal scale, and the subsequent level of ability (or visual difficulty in this case) that a respondent would need to be most likely to answer at a certain response level on the ordinal scale (reported as their beta-threshold, Table 2) [9, 28].

For example, in the IND-VFQ-33 difficulty scale (ranging from (1) “not at all”, to (5) “cannot do this because of my sight”), responses are sequentially dichotomized such that initially, ***k =*** **1** defines one group, and ***k =*** **2*,*3*,*4** ***or*** **5** defines the second group – transforming the polytomous ordinal response scale into an “option = 1 vs. option = (2–5, or)” dichotomy. Sequential dichotomies are made for each individual response on the 4- or 5-point ordinal scale [28]. The GRM then models ***P***(***x***_***ip***_ ***≥ k*** | ***θ***_***p***_), which represents the probability of selecting option ***k*** or higher on item ***i***, given the location of person ***p*** along the ***θ*** scale:

$$ {\boldsymbol{P}}_{\boldsymbol{i}\boldsymbol{k}}^{\ast}=\boldsymbol{P}\left({\boldsymbol{x}}_{\boldsymbol{i}\boldsymbol{p}}\mathbf{\ge}\boldsymbol{k}\right)=\frac{\mathbf{\exp}\left[{\boldsymbol{a}}_{\boldsymbol{i}}\left({\boldsymbol{\theta}}_{\boldsymbol{p}}-{\boldsymbol{b}}_{\boldsymbol{k}}\right)\right]}{\mathbf{1}+\mathbf{\exp}\left[{\boldsymbol{a}}_{\boldsymbol{i}}\left({\boldsymbol{\theta}}_{\boldsymbol{p}}-{\boldsymbol{b}}_{\boldsymbol{k}}\right)\right]} $$

Each item’s discrimination parameter ***a***_***i***_ models how well the item discriminates between respondents of low and medium impairment, or between respondents of medium and high impairment. Generally, higher discrimination is better.

An item with higher overall ***b***_***k***_ parameters indicates more severe impairment (or difficulty). Note that if an item has ***k*** response options, only ***k −*** **1** severity parameters are estimated, which we label ***b***_**2**_ through ***b***_**4**_ or ***b***_**5**_***.*** The model assumes that when a person’s level of impairment equals the ***b***_**2**_ parameter, they are equally likely to endorse response category 2 or higher (i.e. categories 2, 3, 4, or 5) [28] as to endorse categories lower than 2 (i.e. category 1).

IRT additionally calculates beta-thresholds representative of individual item difficulty at differing levels of participant ability. For example, consider two theoretical items which have beta-2 thresholds of 0.5 and 0.8 respectively. For item 1, at *θ* = 0.5, respondents have a 50% chance of endorsing category 2 or higher versus categories lower than 2. For item 2, this threshold is reached when *θ* = 0.8. Thus, as regards to this threshold, item 2 is more difficult, and endorsing category 2 or higher indicates a higher level of visual impairment than the same endorsement for item 1 does.

Items demonstrating either poor discrimination or poor separation of the thresholds are usually removed from further analyses. The GRM then uses the adjusted item discriminatory ability and difficulty calculations of retained items to impute new ‘visual disability’ and cumulative factor scores for individuals.

### Differential item functioning

As part of IRT analyses, a final check on psychometric purity is conducted by checking for Differential Item Functioning (DIF). DIF occurs when the item discrimination and difficulty parameters differ among sociodemographic subgroups even when they are equated on the relevant ability measures. This creates potential measurement biases in favor of one sociodemographic subgroup over another at particular levels of dis/ability [8, 14, 30, 31]. In its simplest form, two groups at a time are investigated for DIF: a reference group (baseline, against which comparisons are made), and a focal group (the population in which DIF is suspected) [30]. We investigated DIF on six dichotomized subgroups; age (< 75 years old vs > 75 years old), gender (male vs female), education (any schooling vs no schooling), housing (pays independently vs financially assistance/subsidized), diabetes (yes vs no), and self-reported depression (categorized using the PHQ-9 questionnaire [32] as either none-mild vs moderate-severe symptoms of depression, using the recommended cut-point of 10 points used for screening for features of depression [32]).

Both uniform and non-uniform DIF analyses were undertaken. Uniform DIF (the constrained model) assumes that only the difficulty parameters differ in the focal group, and so the same direction of bias is present at any level of ability, consistently in favor of the reference group or the focal group. Non-uniform DIF (unconstrained model) assumes that both the difficulty *and* severity parameters differ in the focal group [31]so that the extent of bias in comparing the reference to the focal group is conditional on the level of ability and may even reverse at one level compared to another.

While various statistical approaches for detecting DIF have been developed and researched [33], the ordered logistic DIF procedure is particularly flexible and accurately computes parameter covariance matrices when the IRT model is equated across groups [34, 35]. This approach tests the null hypothesis that the ability differentiation is equal across the entire theta-continuum (the absence of uniform DIF) and the null hypothesis that the item discrimination is equal between each demographic subgroup (the absence of non-uniform DIF). An IRT likelihood-ratio DIF approach was used for the present study, as previously cited [8, 29]. Examining DIF involves multiple tests, and we used the Benjamini-Hochberg (or “false discovery rate”, FDR) procedure to correct the *p*-value thresholds for multiple testing. This correction is the most powerful correction available, meaning that it should not fail to reject a test when significant DIF actually exists. In contrast, other adjustments like the Bonferroni correction are too conservative, meaning that they may fail to flag significant DIF [36].

When DIF is substantial and cannot be ignored, possible solutions include removing or re-writing the item [8] or separate estimation of the item parameters for subgroups; subsequently using those parameters to estimate the person parameter [8, 37]. In the present analysis, assessment of the magnitude in difference in cumulative factor scores between the two subgroups on which there was significant DIF, and the substantive importance/nontriviality of this difference, was used to determine whether to remove the item from the questionnaire.

### Other statistical analyses

#### Goodness of fit

Factor analysis fits are usually followed with various “goodness of fit” indices. We employed two commonly employed such indices: (1) the root mean square error of approximation (RMSEA) which is basically an index of discrepancy between the covariance matrix predicted by the hypothesized model and empirical covariance matrix, and is considered acceptable if < 0.05; (2) the Tucker-Lewis index (TLI) which basically locates the covariance matrix predicted by the hypothesized model on a continuum of that of a null independence model and the empirical matrix, where values > 0.95 are considered good.

#### Missing data

We removed items with over 25% missing responses, as we believed this could indicate that the respondents either did not understand the item or the item was not relevant to them. Pairwise polychoric correlations were then calculated on the remaining items in preparation for exploratory factor analysis.

Stata version 16 (StataCorp LP, College Station, TX) and the R package *lavaan* was used for analyses [38]. 95% confidence intervals are presented where appropriate.

## Results

### Patient demographics

One thousand one hundred eighty-two participants from the HOMES study were originally considered for the present study cohort [18]. Of these, 98 were excluded due to cognitive deficit, and a further 217 medical conditions precluding participation; leaving 867 participants eligible for the present study cohort. Of these, 683 were classified as having normal vision, and 184 were classified as visually impaired. Age and sex were similar between those with visual impairment vs those with normal vision (75 vs 74 years old, and 61.4% vs 62.1% female, respectively) (*p* > 0.05 for both). Those with visual impairment were significantly less likely to have achieved education beyond high school (14.7 vs 24.2%), less likely to independently pay for their housing (35.3 vs. 42.2%), less likely diabetic (21.7 vs. 32.7%) and more likely to have severe depression (21.2 vs. 7.6%) (*p* < 0.05 for all) (Table 1).

**Table 1:** Baseline patient characteristics (*n* = 867)

	Normal vision (***n*** = 683)	Visual Impairment (***n*** = 184)
**Age (mean, (SD))**	74 (8.09)	75 (8.83)
**Female (n, (%))**	424 (62.1)	113 (61.4)
**Education (n, (%))**		
- **< High School**	70 (10.3)	46 (25)
- **High School**	448 (65.6)	111 (60.3)
- **> High School**	165 (24.2)	27 (14.7)
**Housing (n, (%))**		
- **Fully subsidized**	92 (13.5)	35 (19)
- **Partially subsidized**	303 (44.4)	84 (45.7)
- **Independently paid**	288 (42.2)	65 (35.3)
**Diabetes (n, (%))**	223 (32.7)	40 (21.7)
**Depression (n, (%))** ^**a**^		
- **None-Mild**	546 (79.9)	126 (68.5)
- **Moderate**	85 (12.5)	19 (10.3)
- **Severe**	52 (7.6)	39 (21.2)

Key: (a) PHQ-9 depression score; categorized as either none-mild (sum score 0–9), moderate (sum score 10–19), or severe (sum score 20–27) depression

### Unrotated and rotated (Promax oblique) factor analysis

**Fig. 1:**
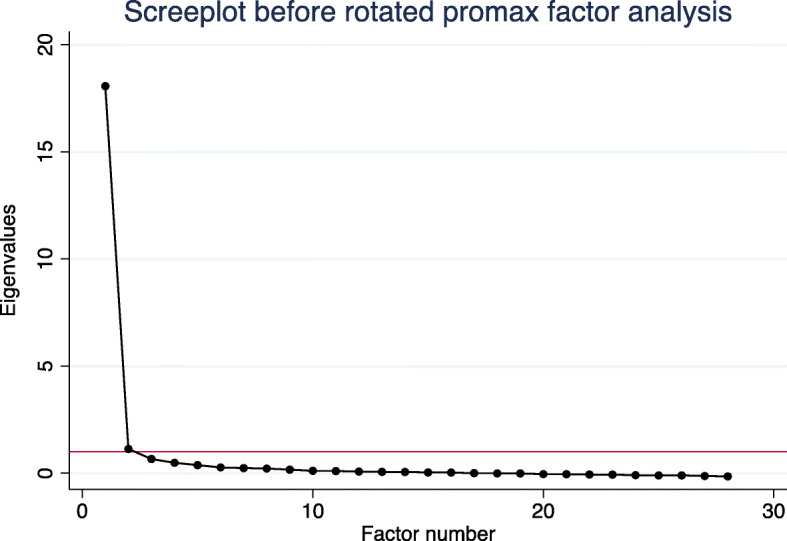
Screeplot before rotated promax oblique factor analysis. Key: y-axis reference line at the minimum eigenvalue threshold of 1.0, displayed

**Fig. 2:**
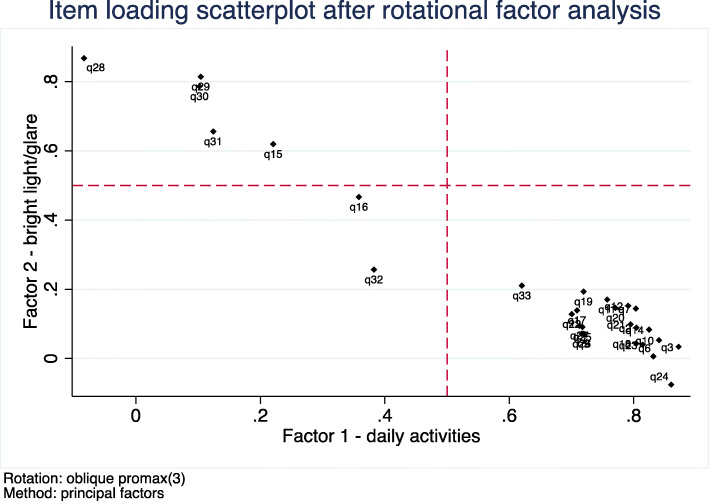
Item loading scatterplot after rotational factor analysis. Key: x-axis and y-axis reference line at the minimum item loading threshold of 0.5 on either factor, displayed. Factor 1 and factor 2 correlation = + 0.68

**Fig. 3:**
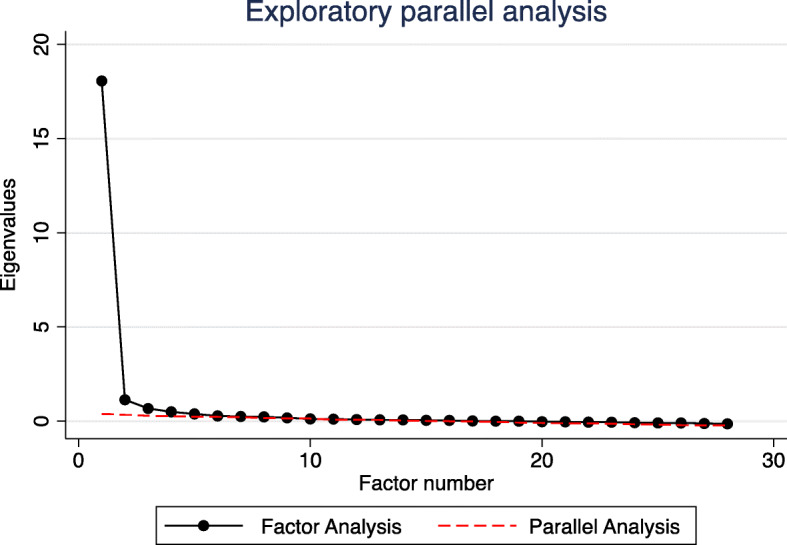
Exploratory parallel analysis. Key: Dashed line represents the 95th percentile of the distribution of respective counterparts produced by random permutations of the data

Exploratory unrotated factor analysis identified one factor with a particularly high eigenvalue (Factor 1, eigenvalue 18.1), and a second factor with a lower eigenvalue still above our eigenvalue threshold (Factor 2, eigenvalue 1.1). The remaining 31 factors all had eigenvalues < 0.6. We then conducted an oblique Promax factor rotation (Fig. 1) for a 2-factor solution. Most items loaded well on one or the other of the two factors identified (Fig. 2, Table 2). The first factor appeared to describe impairments in daily activities and function (thereafter labelled Factor 1: “Daily Activities”). The second factor described impaired ability to tolerate bright light or glare (thereafter labelled Factor 2: “Bright Lights”). The estimated correlation between the two factors was + 0.68; thus, results indicate two distinct but moderately positively related factors. Items 16 (“do you have trouble seeing inside after being outside in sunlight”) and 32 (“does light seem like stars”) loaded poorly onto both factors and were subsequently removed before IRT (Fig. 2). An exploratory parallel analysis suggested a total of 4 discrete factors may lay above the threshold of random permutations of the data (Fig. 3). However, after rotation, the suggested 4 factor solution had uninterpretable third and fourth factors with no strong clinical associations between items uniquely loading on each factor, and items had substantially weaker loadings on their primary factors. Subsequently, these additional third and fourth factors were not considered for further analyses.

**Table 2:** Identified factors from rotated promax factor analysis with individual item loading

	Factor 1: Daily Activities	Factor 2: Bright Lights
*1: Climbing stairs (Item removed (missing > 25%))* ^*a*^		
2. Making out bumps in the road	**0.80** ^**b**^	*0.09* ^*c*^
3. Seeing animals or vehicles walking	**0.87**	*0.04*
*4: Going to functions like weddings (Item removed (missing > 25%))*		
*5: Finding way in new places (Item removed (missing > 25%))*		
6. Going out at night	**0.83**	*0.01*
7. Finding way around indoors	**0.80**	*0.14*
*8: Climbing on or off buses (Item removed (missing > 25%))*		
9: Recognizing people from a distance	**0.72**	*0.07*
10. Recognizing a person near you	**0.84**	*0.05*
11. Locking or unlocking the door	**0.76**	*0.17*
12. Doing your usual work at home	**0.79**	*0.15*
*13: Doing work to your usual standard (Item removed (missing > 25%))*		
14. Searching for things at home	**0.82**	*0.08*
15. Seeing outside in bright sunlight	*0.22*	**0.62**
*16. Seeing inside after being out in sunlight (item removed (poor loading < 0.5))* ^*d*^		
17. Seeing differences in color	**0.71**	*0.14*
18. Differentiating between money	**0.80**	*0.04*
19. Going to the toilet	**0.72**	*0.19*
20. Seeing objects fallen in your food	**0.77**	*0.15*
21. Seeing container level when pouring	**0.79**	*0.10*
22: Frightened going out at night	**0.70**	*0.13*
23. Enjoy social functions less	**0.81**	*0.04*
24. Ashamed that you can’t see	**0.86**	*−0.08*
25: Become a burden on others	**0.72**	*0.09*
26: Frightened to lose remaining vision	**0.72**	*0.07*
27: Do you have reduced vision	**0.71**	*0.09*
28. Dazzled in bright light	*−0.08*	**0.87**
29. Blurry vision in the sunlight	*0.10*	**0.81**
30. Bright light hurt your eyes	*0.10*	**0.79**
31. Vehicle light makes you close eyes	*0.12*	**0.66**
*32: Does light seem like stars (Item removed (poor item loading < 0.5))*		
33: Do you have blurred vision	**0.62**	*0.21*

Key: (a) item removed due to high missingness; (b) unique item factor loading > 0.5 threshold (bolded); (c) unsubstantial item factor loading < 0.5 (un-bolded, italicized); (d) item loaded poorly onto either factor < 0.5

### Item response theory (graded response models)

Table 3 presents the results of the Item Response Theory Graded Response Model analysis. All 21 remaining items loading onto Factor 1 had a high discrimination > 2.0. All 5 remaining items loading onto Factor 2 had a high discrimination of > 2.0 except item 31 (discrimination = 1.9).

**Table 3:** Final shortlist of items with discrimination and severity parameters

	Discrimination (CI)	B2 threshold	B3 threshold	B4 threshold	B5 threshold
**Factor 1: Daily Activities**					
**2. Making out bumps in the road**	2.9 (2.5–3.4)	0.5 ^a^	1.4	1.7	2.7
**3. Seeing animals or vehicles walking**	3.6 (3.0–4.2)	1.1	1.6	1.9	2.8
**6. Going out at night**	2.5 (2.1–2.8)	0.8	1.5	1.8	2.2
**7. Finding way around indoors**	4.0 (3.2–4.7)	1.4	2.0	2.3	3.4
**9. Recognizing people from a distance**	2.1 (1.8–2.4)	0.0	0.9	1.1	1.5
**10. Recognizing a person near you**	3.4 (2.8–4.1)	1.4	2.1	2.4	2.9
**11. Locking or unlocking the door**	3.4 (2.8–4.0)	1.2	1.8	2.2	2.6
**12. Doing your usual work at home**	3.6 (3.0–4.2)	1.1	1.7	2.0	2.9
**14. Searching for things at home**	3.3 (2.8–3.8)	0.9	1.6	1.9	2.7
**17. Seeing differences in color**	2.5 (2.1–2.9)	1.0	1.8	2.1	2.7
**18. Differentiating between money**	2.7 (2.3–3.1)	0.8	1.6	1.9	2.4
**19. Going to the toilet**	3.2 (2.6–3.8)	1.3	1.9	2.2	3.6
**20. Seeing objects fallen in your food**	3.2 (2.7–3.7)	1.0	1.6	1.9	2.3
**21. Seeing container level when pouring**	3.1 (2.6–3.5)	1.0	1.7	1.9	2.5
**22. Frightened going out at night**	2.2 (1.9–2.6)	0.8	1.4	1.6	–
**23. Enjoy social functions less**	2.7 (2.3–3.2)	1.1	1.5	1.7	–
**24. Ashamed that you can’t see**	2.4 (2.0–2.9)	1.4	1.9	2.3	–
**25. Become a burden on others**	2.2 (1.8–2.6)	1.3	1.8	2.0	–
**26. Frightened to lose remaining vision**	2.0 (1.7–2.3)	0.8	1.4	1.7	–
**27. Do you have reduced vision**	2.2 (2.0–2.5)	−0.1	1.3	1.8	–
**33. Do you have blurred vision**	2.2 (1.9–2.5)	0.2	1.3	1.9	–
**Factor 2: Bright Lights**					
**16. Seeing inside after being out in sunlight**	2.2 (1.8–2.5)	0.3	1.4	1.8	2.8
**28. Dazzled in bright light**	2.5 (2.1–2.9)	0.4	1.4	1.8	–
**29. Blurry vision in the sunlight**	3.5 (2.9–4.2)	0.2	1.2	1.7	–
**30. Bright light hurt your eyes**	2.9 (2.4–3.3)	0.5	1.2	1.8	–
**31. Vehicle light makes you close your eyes**	1.9 (1.6–2.2)	−0.4	0.9	1.3	–

Key: (a) beta-threshold representing visual difficulty theta level at which it becomes more likely for participant to choose option 2 vs option 1 on the 5-point Likert scale

The item difficulty parameters (B2 to B5 thresholds) reflect the range of underlying participant ability for each Factor at which it becomes more likely to select the difficulty option higher on the 4- or 5-point ordinal scale (Table 3). All items within each factor showed good separation between difficulty thresholds, allowing for good differentiation of participant ability (or visual difficulty) for any given item.

### Differential item functioning

**Fig. 4:**
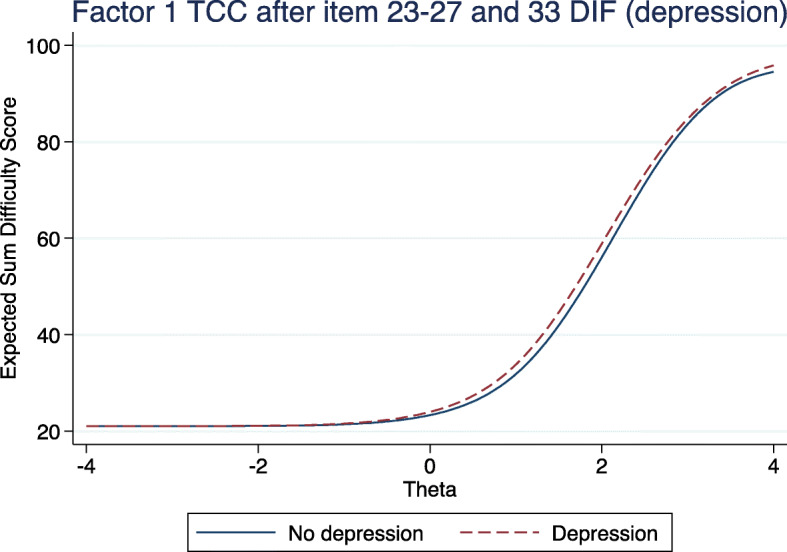
Test characteristic curve (TCC) for factor 1 (DIF on depression for items 23–27, and 33)

Table 4 presents the items demonstrating statistically significant DIF for Factor 1 and Factor 2. For Factor 1, depression was the only baseline variable causing DIF on six items in total (items 23–27 and item 33); all demonstrating significant uniform DIF except item 24, which was non-uniform (note the different discrimination (or sigmoid slope) by depression, for item 24). There was no substantive difference in beta thresholds for any of the six items by depression (Table 4) and no substantive difference in expected item score for items loading Factor 1, by depression at baseline (see Fig. 4, outlined below). For Factor 2, only item 31 demonstrated significant uniform DIF on gender. Similarly, the beta thresholds did not exhibit major differences between gender (Table 4), and the expected total score was very similar between genders at baseline.

**Table 4:** Items with significant uniform and non-uniform differential item functioning

	Subgroup	Discrimination	B2 threshold	B3 threshold	B4 threshold	B5 threshold	***P***-value (BH) ^**a**^	***P***-value (DIF) ^**b**^
**Factor 1: Daily Activities**								
**Uniform Differential Item Functioning**								
**23: Enjoy social functions less**	No depression	2.4	1.5 ^c^	2.0	2.3	–	0.008	< 0.001
	Depression	2.4	1.3	1.7	2.0	–		
**25: Become a burden on others**	No depression	1.8	2.0	2.8	2.9	–	0.004	< 0.001
	Depression	1.8	1.5	2.0	2.2	–		
**26: Frightened to lose remaining vision**	No depression	1.6	1.2	2.1	2.4	–	< 0.001	< 0.001
	Depression	1.6	0.8	1.5	1.9	–		
**27: Do you have reduced vision**	No depression	2.0	0.1	1.7	2.2	–	0.006	< 0.001
	Depression	2.0	0.0	1.5	2.2	–		
**33: Do you have blurred vision**	No depression	2.0	0.4	1.7	2.4	–	0.006	< 0.001
	Depression	2.0	0.4	1.5	2.2	–		
**Non-Uniform Differential Item Functioning**								
**24: Ashamed that you can’t see**	No depression	2.6	1.9	2.4	2.7	–	0.01	< 0.001
	Depression	1.6	1.5	2.3	2.9	–		
**Factor 2: Bright Lights**								
**Uniform Differential Item Functioning**								
**31: Vehicle light makes you close eyes**	Male	1.8	−0.7	0.8	1.3	–	0.003	=0.001
	Female	1.8	−0.5	1.0	1.4	–		

Key: (a) Benjamini-Hochberg adjusted p-value significance threshold; (b) unadjusted raw p-value (results of the likelihood ratio test (uniform or non-uniform DIF vs base model)); (c) beta-threshold representing the individuals’ visual difficulty theta level at which it becomes more likely for them to choose that option on the 4- or 5-point Likert scale, i.e. at visual difficulty theta level 1.5, it becomes more likely for an individual without depression to choose option 2 rather than option 1 on the 4-point scale

**Fig. 5:**
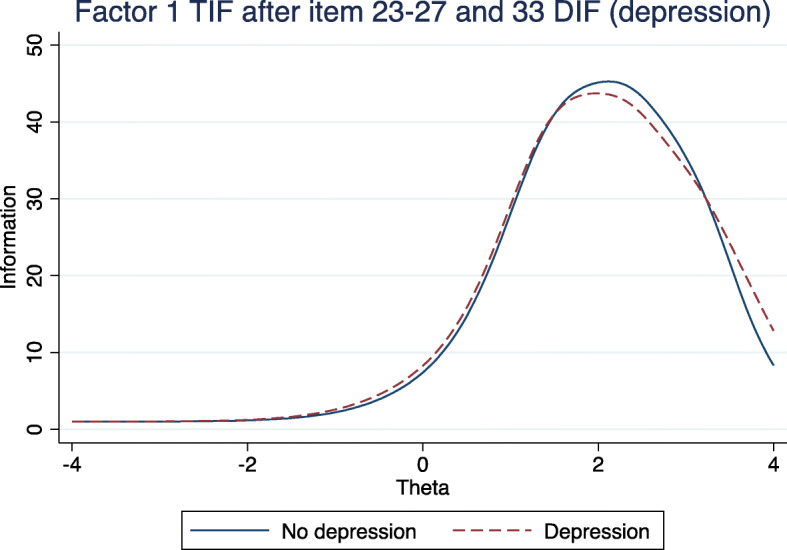
Total information curve (TIF) for factor 1 (DIF on depression for items 23–27, and 33)

Figure 4 demonstrates the expected total score by depression for Factor 1, allowing all 6 items identified above to have DIF. As demonstrated, the expected Factor 1 score was almost identical at all levels of visual impairment regardless of depressive symptoms at baseline; those with depression scoring fractionally higher sum difficulty scores. Figure 5 similarly demonstrates a similar level of total visual difficulty information available at all levels of theta ability between depression at baseline.

### Goodness of fit

The RMSEA for our first dimension of overall visual impairment (Factor 1) was 0.038, indicating close fit and that it is essentially unidimensional despite including some psychosocial items. The second dimension of glare (Factor 2) had a lower RMSEA, 0.102, higher than the usually accepted cutoff of 0.05. However, our proposed model as a whole had RMSEA of 0.037. In addition, the TLI, another goodness of fit index, was well over the accepted criterion of 0.95 for each individual dimension and the model as a whole (overall impairment 0.989, glare 0.980, full model 0.987). Fitting Gupta’s original model to our data, we obtained acceptable RMSEAs for the dimensions of visual impairment and psychosocial symptoms (0.037 and 0.043 respectively), but an even higher RMSEA for their last dimension of visual symptoms (0.148). This subscale overlaps with our proposed glare dimension, but contains symptoms not related to glare. In any case, the TLI values for each dimension and the overall scale were high with the original structure as well (visual impairment 0.992, psychosocial 0.995, visual symptoms 0.926, overall model 0.982).

### Recalibration, final item reduction

As a result of near-identical sum Factor 1 and Factor 2 scores at all levels of difficulty between either depression or gender at baseline (respectively) after DIF analysis, no further item recalibration or reduction was undertaken.

## Discussion

The purpose of the present study was to psychometrically evaluate the IND-VFQ-33 ophthalmology questionnaire with contemporary psychometric validation techniques not previously used, clarifying latent traits being assessed and which items substantively loaded on each trait (through Factor Analysis), ensuring adequate discrimination and differentiation (through IRT), and excluding potential bias between demographic subgroups (through DIF analysis).

Of the 33 items in the IND-VFQ-33, we removed a further 7 after psychometric validation; 5 items initially removed because of high frequency of missing values (potentially due to wording ambiguity or poor relevance to a residential aged-care population), and thereafter removing item 32 (“does light seem like stars”) and item 16 (“do you have trouble seeing inside after being outside in sunlight”) for poor loading onto either of the two factors. We subsequently present 26 remaining items, all of which demonstrate good discrimination and differentiation after IRT (GRM) validation, and load well onto one of the two clinically distinct factors; Factor 1 (“Daily Activities”) and Factor 2 (“Bright Lights”).

Importantly, 6 items in Factor 1 exhibited DIF in respondents with significant current depressive symptoms. However, as illustrated in Fig. 4 the overall impact on the cumulative factor score is minimal: respondents with depression had almost identical expected scores across all potential levels of visual impairment. While there is a method to formally test if the difference in the expected scores is different between two groups [30], we elected not to do this because the difference is much less than one raw score point at any level. Additionally, in respondents with depression, the estimated severity thresholds did not differ substantially from the reference group. Similarly, there is detectable DIF for gender on one question in Factor 2, but it does not make a substantial difference to the expected cumulative Factor 2 score. Thus, we presently argue that the instrument as a whole can be treated as not having DIF.

Notably in the present study, our initial unrotated factor solution indicated the presence of one strong latent factor (suggested by a particularly prominent first eigenvalue) and a weaker but still evident additional factor (an eigenvalue marginally above our 1.0 value threshold for acceptance and statistically significant by the parallel analysis test). After rotation to a more meaningful 2 factor solution, there was indeed a moderately-high positive correlation between Factor 1 and Factor 2, suggesting that there may be one higher-order latent trait assessed by the IND-VFQ-33 instrument as a whole, but the decision was made here to include two separate factors which were still indicated as distinct. This was in part because of the unique, strong loadings onto Factor 2 for a set of items all of which loaded poorly onto Factor 1 (particularly items 28–30), and which were clinically unique in their description of symptoms relating to bright light, which none of the questions preferentially loading on Factor 1 described.

The two distinct factors found in the present study might be compared to two of the original domains outlined by Gupta and colleagues; “general function” and “visual symptoms”. Here, we demonstrate that two items originally representative of “visual symptoms” by Gupta and colleagues (item 27 (“do you have reduced vision”) and item 33 “do you have blurred vision”)), instead load preferentially with Factor 1, “Daily Activities”. Notably, a third distinct factor representative of the remaining domain originally outlined (“psychosocial impact”) was not found here. The five items originally pertaining to that third “psychosocial impact” factor (items 22–26) presently all loaded well onto Factor 1, without demonstrating their own unique sub-scale. While definitive conclusions cannot be drawn to explain this, the relatively high prevalence of depression may have confounded participants’ responses to psychosocial impact items.

In contrast to our study, the Rasch-validated analysis of the questionnaire by Gothwal and colleagues discarded 13 of the 33 items, and two of the original three questionnaire domains, for not adequately demonstrating Rasch properties; subdividing the only remaining domain (“general function”) into two sub-scales and substantially reducing the total questionnaire content considerably. As aforementioned, the Rasch model is a very restrictive model. Items not meeting the restrictions are discarded, which led to Gothwal et al. eliminating many more items than we did here. Additionally, Rasch validation is not necessarily designed to detect multidimensionality of questionnaires. Indeed, Harvey argued that it may be better to start with less restrictive psychometric models [39], such as the GRM.

### Strengths and limitations

Strengths of the study include the relatively large total sample size, and the relatively high response rate for most items. The clinical assessments and interviews were done within the residential aged-care homes to ensure comfort and convenience for all participants, and contemporary psychometric validation techniques that have not previously been applied to the IND-VFQ-33 allowed for accurate psychometric testing. With globally aging populations, a validated instrument to assess the burden of visual impairment in the elderly is imperative. Through contemporary psychometric validation methods, we here describe how this visual survey tool might be better used for elderly populations; important in eye care planning, resource allocation and directing future research. Possible limitations of the current study include potential inaccuracies associated with self-reported data (i.e. reported level of visual difficulty and reported depression symptoms), a limitation faced by all studies using self-reported data. The fact that the cohort were exclusively residential aged-care participants from the HOMES study (with an average age of 74–75 years old) limits the generalizability of current findings, making it potentially less applicable to a general community population. Indeed, the high missingness for 5 of the original items (which were subsequently removed from further analyses) may have been because those tasks are not routinely performed by aged care residents (i.e. climbing stairs or climbing onto/off buses). The relatively high proportion of participants with depression may have partially contributed toward our not finding a clinically distinct domain for psychosocial impact. While the RMSEA for our proposed Factor 2 (bright lights) dimension was > 0.10 (similarly to the RMSEA for the equivalent dimension in the original model, and potentially indicative of poor fit), we note that the items we retained for Factor 2 form a distinct clinical entity and are more homogeneous than the items proposed in the original factor. In addition, the high TLI of > 0.98 for this dimension and for our model as a whole in our CFA analysis are supportive of its inclusion – also supported elsewhere [40]. Finally, the generalizability of our findings is also limited by the exclusion of participants with impaired cognition or medical comorbidities precluding participation.

## Conclusion

Here, we applied Factor Analysis, Item Response Theory, and Differential Item Functioning psychometric-validation techniques to the IND-VFQ-33 questionnaire. We identified 2 discrete (but somewhat correlated) factors with 26 uniquely-loading items. These 2 factors are clinically representative of difficulty performing daily activities and experiencing difficulty due to bright light or glare, respectively. Our modified 26-item scale may be useful in evaluating symptomatic disease progression or response to treatment, particularly in an older aged population in India.

## Data Availability

The dataset generated and analysed during the current study are not publicly available due to confidentiality and consent taken from patients at the time of data collection. The data may be made available from the corresponding author upon reasonable request.

## Abbreviations

IND-VFQ-33: Indian visual field questionnaire
IRT: Item response theory
GRM: Graded response model
DIF: Differential item functioning
FDR: False discovery rate
HOMES: Hyderabad ocular morbidity in the elderly study
CFA: Confirmatory factor analysis
RMSEA: Root mean squared error of approximation
TLI: Tucker lewis index
